# Dietary patterns and internalizing symptoms in children and adolescents: A meta-analysis

**DOI:** 10.1177/00048674211031486

**Published:** 2021-07-27

**Authors:** Laura Orlando, Katarina A Savel, Sheri Madigan, Marlena Colasanto, Daphne J Korczak

**Affiliations:** 1 Department of Psychiatry, Faculty of Medicine, University of Toronto, Toronto, ON, Canada; 2 Department of Human Biology, University of Toronto, Toronto, ON, Canada; 3 Department of Psychology, Faculty of Arts, University of Calgary, Alberta Children’s Hospital Research Institute, Calgary, AB, Canada; 4 Applied Psychology and Human Development, University of Toronto, Toronto, ON, Canada; 5 Department of Neuroscience and Mental Health, The Hospital for Sick Children, Toronto, ON, Canada; 6 Department of Psychiatry, The Hospital for Sick Children, Toronto, ON, Canada

**Keywords:** Child and adolescent psychiatry, mood and anxiety disorders, dietary patterns

## Abstract

**Context:**

Studies of child and adolescent internalizing symptoms and dietary pattern have produced mixed results.

**Objectives:**

To quantify the association between dietary patterns and internalizing symptoms, including depression, in children and adolescents.

**Data sources:**

Embase, PsycINFO, MEDLINE, Web of Science and Cochrane up to March 2021.

**Study selection:**

Observational studies and randomized controlled trials with mean age ⩽ 18 years, reporting associations between diet patterns and internalizing symptoms.

**Data extraction:**

Mean effect sizes and 95% confidence intervals were determined under a random-effects model.

**Results:**

Twenty-six studies were cross-sectional, 12 were prospective, and 1 used a case-control design. The total number of participants enrolled ranged from 73,726 to 116,546. Healthy dietary patterns were negatively associated with internalizing (*r* = –0.07, *p* < 0.001, 95% confidence interval [–0.12, 0.06]) and depressive symptoms (*r* = –0.10, *p* < 0.001, 95% confidence interval [–0.18, –0.08]). Effect sizes were larger for studies of healthy dietary patterns and internalizing and depressive symptoms using self-report versus parent-report measures, as well as in cross-sectional studies of healthy dietary patterns and depression compared to prospective studies. Unhealthy dietary patterns were positively associated with internalizing (*r* = 0.09, *p* < 0.001, 95% confidence interval [0.06, 0.14]) and depressive symptoms (*r* = 0.10, *p* < 0.01, 95% CI [0.05, 0.17]). Larger effect sizes were observed for studies of unhealthy dietary patterns and internalizing and depressive symptoms using self-report versus parent-report measures.

**Limitations:**

A lack of studies including clinical samples and/or physician diagnosis, and a paucity of studies in which anxiety symptoms were the primary mental health outcome.

**Conclusion:**

Greater depression and internalizing symptoms are associated with greater unhealthy dietary patterns and with lower healthy dietary intake among children and adolescents.

## Introduction

One in five children and youth worldwide report mental health problems (The Mental Health of Children and Youth in Ontario: A Baseline Scorecard, 2015; World Health Organization, 2003). In particular, internalizing problems, defined as symptoms of depression, anxiety and emotional problems, are frequently reported symptoms among those under the age of 18 years, with increasing proportions of youth endorsing these symptoms over time (Boak et al., 2014). The early identification and treatment of internalizing disorders, such as depression and anxiety, during the child and adolescent years may be critical to prevent ongoing morbidity and mortality from these conditions across the lifespan.

Interest in the role nutrition plays in mental health, known as nutritional psychiatry, has grown in recent years (Adan et al., 2019). Childhood is a time of rapid brain growth, and the impact of specific nutrients, such as dietary omega 3 fatty acids, on cognition has been well-established (Gómez-Pinilla, 2008). There are several hypothesized mechanisms which could explain the link between dietary patterns and internalizing symptoms, including biological explanations (inflammation, gut-brain axis and Brain-Derived Neurotrophic Factor [BDNF]) as well as psychosocial factors. There is also a wide range of ways in which dietary intake (i.e. food frequency questionnaires, diet history questionnaires) and subsequently dietary patterns, including diet quality indices, is characterized in research. In addition, there has been growing interest in considering overall dietary patterns as opposed to single nutrients. This broader approach may help us to better understand associations between dietary patterns and both physical and mental health disease risk compared with the single nutrient approach (Tapsell et al., 2016; World Cancer Research Fund International, 2018). This is because single nutrients and other dietary components are not ingested in isolation, and it is likely that synergistic and potentially antagonistic effects of dietary components influence health and disease risk (Tapsell et al., 2016). Consequently, understanding the effects of overall dietary pattern may have important implications for designing effective interventions to promote health, including mental health.

A recent meta-analysis of 16 studies (*n* = 45,825) of adults found that dietary interventions reduced depressive symptoms (Firth et al., 2019), suggesting dietary patterns may be an important modifiable risk factor for depression. In this recent meta-analysis of dietary interventions, ‘whole-of-diet’ interventions were included, as opposed to those focused on individual foods or nutrients, so studies which looked at global measures of diet were selected. While no meta-analysis of literature focused on children and youth has been conducted to date, systematic reviews that have examined these associations in young people have suggested a possible positive association between healthy dietary patterns and improved mental health, as well as unhealthy dietary patterns and poorer mental health (Khalid et al., 2016; O’Neil et al., 2014). In these studies, and consistent with previous research, a ‘healthy’ diet was defined as one with a greater intake of nutrient dense foods, and an ‘unhealthy’ diet was noted to have more highly processed foods and sugars, refined carbohydrates and saturated fats. Thus, an ‘unhealthy’ diet could be considered as less healthy on the spectrum of dietary patterns. Many studies included in these reviews refer to this less healthy dietary pattern as ‘Western’ in nature (Khalid et al., 2016; O’Neil et al., 2014). That said, empirical findings across the literature have been inconsistent. While clinical best-practice guidelines outline the importance of a healthy eating pattern to improve depression in children and youth (National Institute for Health and Care Excellence, 2018), conflicting data present challenges to clinicians attempting to counsel children and families on specific recommendations for adjustment to diet quality to improve depressive symptoms.

The primary objective of this meta-analysis was to quantify the association between dietary patterns and internalizing problems, defined as symptoms of depression, anxiety and emotional problems, among children and youth under the age of 18 years. Depressive symptoms were highlighted given the abundance of studies looking specifically at these symptoms and diet. Individuals under the age of 18 were also chosen as it is important to examine if the association seen in adulthood between internalizing problems and dietary pattern is present earlier in life, highlighting a potentially modifiable risk factor in the development of potentially lifelong mental health symptoms. To our knowledge, this is the first meta-analysis to examine this association in children and youth. Our secondary objective was to identify potential moderating factors, as these can be particularly informative for clinical interventions, public health policies, and programs related to the promotion of healthy eating and mental health for children and families.

## Methods

### Search strategy

**Figure 1.**
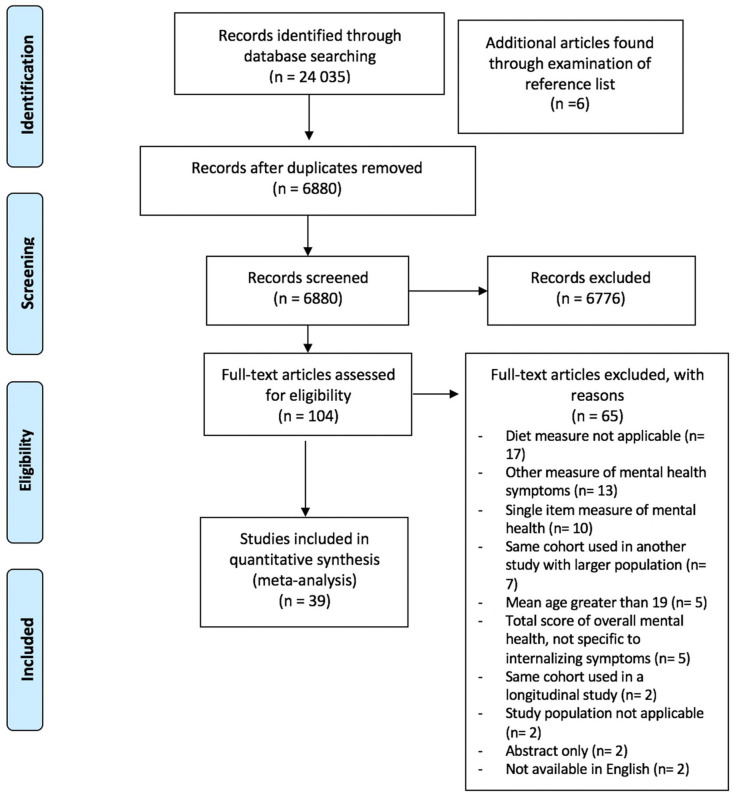
PRISMA flow diagram of literature search and study selection.

Published studies on internalizing symptoms and dietary patterns were identified by searching the following databases: Embase, PsycINFO, MEDLINE, Web of Science and Cochrane on 20 March 2021. The search was limited to English language articles using keywords related to ‘children or youth’, ‘depression or anxiety or internalizing disorders’ and ‘diet or nutrition’. These search terms were then combined with the Boolean ‘AND’. References of included studies were also searched for additional studies meeting inclusion criteria (see PRISMA Flow Figure 1). No other sources of information were reviewed. The review protocol was registered with the PROSPERO database with reference number CRD42020160405 and is available from https://www.crd.york.ac.uk/prospero/display_record.php?ID=CRD42020160405.

### Study inclusion and exclusion criteria

The following inclusion criteria were applied: (1) full-text articles available in English; (2) cross-sectional or longitudinal study design; (3) participant mean age ⩽ 18 years; (4) association between diet and depression and anxiety symptoms as an outcome was provided; and (5) overall dietary pattern, as measured by self-report food frequency questionnaires or another source, was provided. Articles that assessed dietary patterns using only single food items or food groups (i.e. sugar-sweetened beverages only), or that examined individual micronutrients or eating behaviors (i.e. binge eating) exclusively, or employed single-item measures of depression and anxiety symptoms or disorders, were excluded (see PRISMA Flow Figure 1).

When the same dataset was used in multiple publications, only one study was included. The included study was selected based on the following protocol: if a dataset was used in both cross-sectional and longitudinal studies, the latter was included to maximize the number of prospective studies included in the sample, given the abundance of cross-sectional studies in the literature and the stronger conclusions that can be drawn from longitudinal data. If a single dataset was used in multiple different studies with the same study design, the publication with the largest sample size was included (Higgins et al., 2003).

### Data extraction

Titles and abstracts were screened (L.O.), and full-text articles were further assessed for eligibility in an independent manner (L.O. and K.A.S.). Data extraction of full-text articles was performed by two independent coders (L.O. and K.A.S.). A standard coding form was used to collect information on study and sample characteristics. Data were extracted on categorical moderators including study design (cross-sectional or longitudinal), respondent (self-report or parent-report), dietary measure and validity, and type of mental health measure. Data were collected on continuous moderators including study year, sample size, percent boys in the sample, percent of the sample that presented with internalizing symptoms or met criteria for a disorder diagnosis, percent overweight and obese, and percent high socioeconomic status. Adjusted effect sizes were extracted when provided.

In some studies, sample data were stratified by diet quality (i.e. diet quality quartiles) and/or by internalizing symptom status (i.e. positive screen for internalizing symptoms). Results were extracted from the extreme end of the diet quality measure (i.e. most or least healthy and/or unhealthy) and/or from the highest level of internalizing symptom severity.

### Quality assessment

Study quality was assessed using the Joanna Briggs Institute Critical Appraisal Checklists for cross-sectional, cohort and case-control studies (Joanna Briggs Institute, 2017a, 2017b, 2017c). Assessment was based on the presence or absence of the following: (1) clearly defined inclusion criteria for the sample, (2) detailed description of the study sample and setting, (3) diet measure had been tested for validity, (4) identification of confounding variables, (5) adjustment for confounding variables, (6) validated mental health measure and (7) appropriate statistical analysis and report of relevant data. Prospective study assessment included acceptable attrition rate (less than 20%), and case-control study assessment included appropriate matching of groups. No follow-up time criterion was applied to prospective studies, as all follow-up times were greater than 6 months. Validity of diet and mental health measures was determined by first checking the included article for indication of measure validity or through a search for publications on measure validity when not indicated in the primary article.

### Data synthesis and analysis

Effect sizes were calculated using Comprehensive Meta-Analysis (CMA, 2020) version 3.0 software. Beta values, odds ratios and hazard ratios were entered into CMA. Beta values were entered as correlations into CMA and hazard ratios were entered with *p*-value estimates for one study. All effect sizes were transformed into pooled correlations (*r* values) with 95% confidence intervals (CIs) using random-effects meta-analysis, representing the average association between mental health and dietary pattern across studies. Consistent with recent recommendations for psychological research (Funder and Ozer, 2019), pooled effect size magnitudes are interpreted as small, moderate and large based on *r* values of 0.1, 0.2 and 0.3, respectively.

Heterogeneity of mean effect sizes were assessed using Cochran’s *Q* and *I*^2^ statistics. Cochran’s *Q* is a test of the null hypothesis that the true effect size is shared across studies, and any variance is due to chance (Cochran, 1954). The *I*^2^ statistic is the percentage of total variance across studies due to heterogeneity, as opposed to chance (Higgins et al., 2003). Mixed-effect analyses were used to determine the effect of categorical moderators and meta-regression was used to determine the effect of continuous moderators. Publication bias was assessed using Egger’s regression (Egger et al., 1997) and funnel plots. When asymmetry was indicated, Duval and Tweedie’s (2000) trim-and-fill analysis was used to estimate adjusted mean effect sizes. Outliers were identified using the boxplot function in R version 4.0.2 (R: The R Project for Statistical Computing, 2020).

A wide spectrum of dietary patterns was defined in the literature. Some variables were continuous, while many studies operationalized the terms ‘healthy’ and ‘unhealthy’ based on variables such as the amount of processed or fast foods consumed versus vegetables or complex carbohydrates. During extraction, each measure of diet was identified and summarized individually. To synthesize the information, the measures were then broadly classified as a ‘healthy’ or ‘unhealthy’ dietary pattern. Several publications presented separate results for the association between mental health outcomes and measures of healthy and unhealthy diet quality. For this reason, effect sizes were grouped according to whether the diet variable represented healthy or unhealthy diet quality, and meta-analyses were performed separately for these two groups. In publications where fruit and vegetable intake were distinct independent variables, effect sizes were averaged to produce an overall association between a healthy dietary pattern and mental health outcomes. This was done to fit with the study objective of examining the association between internalizing symptoms and overall dietary pattern, rather than individual foods or food groups.

Meta-analyses were first performed with all internalizing mental health measures (depression, anxiety and emotional symptoms). Effect sizes were pooled if studies reported multiple results (i.e. for depression and anxiety), and if results were presented separately for girls and boys. A separate meta-analysis was performed for studies where the primary mental health outcome was depression, as the majority of included studies focused on depression, rather than anxiety or emotional symptoms.

## Results

The search of online databases yielded 24,035 total records and 6 additional records were found through examination of reference lists (Figure 1). After duplicates were removed, the remaining 6880 articles were screened. Following review of title and abstract, 104 full-text articles remained for full-text data extraction by two independent coders. Thirty-nine articles met full inclusion criteria and were included in the meta-analysis.

### Study and sample characteristics

#### Study characteristics

Study characteristics are presented in Table 1. Twenty-six studies were cross-sectional, 12 were prospective, and 1 used a case-control design. The following total number of participants was included in the analyses: 116,546 with respect to healthy dietary patterns and internalizing symptoms; 77,512 in healthy dietary patterns and depressive symptoms; 116,044 in unhealthy dietary patterns and internalizing symptoms; and 73,736 in unhealthy dietary patterns and depressive symptoms. Participant age ranged from 3.9 to 18 years. With respect to age and as outlined in Table 1, the following classification was found in the cross-sectional studies: 1 study of early and middle childhood (birth–year 8, and year 9–year 12); 4 studies of middle childhood (year 9–year 12); and 22 studies included adolescents (year 13–year 18). Of the prospective studies, there were 3 studies of early childhood, 1 study of middle childhood and 7 studies which consisted of adolescents. To collect dietary information, 37 studies used food frequency questionnaires, 2 of which were administered by trained professionals, 1 study used a 4-day diet diary, and 1 study used a 24-hour recall. Seventeen studies assessed both healthy and unhealthy dietary patterns, while 15 assessed only healthy dietary pattern and 7 assessed only unhealthy dietary pattern. The mental health outcome of interest was depression in 24 studies, depression and anxiety in 3 studies, and emotional symptoms in 12 studies. No study examined anxiety as an outcome variable in the absence of depression. Thirty-seven studies used screening questionnaires to assess mental health (28 self-report, 8 parent-report, 1 teacher-report), and 2 studies used physician diagnosis of an internalizing disorder. Dietary intake was determined using self-report in 30 studies, parent-report in 8 studies and teacher-report in 1 study. For studies reporting prevalence rates, the rate of clinically significant depression symptoms ranged from 10.8% to 33.3%, and the rate of potentially clinically significant emotional symptoms in general ranged from 2% to 62.7%.

**Table 1.** Study characteristics.

Study		Sample			Measures					Results	
Author	Country	Sample size	Age (mean SD)	% boys	Dietary intake	Healthy diet	Unhealthy diet	Mental health	Informant	Mental health characteristics	Significant findings
*Cross-sectional*											
Castillo et al. (2014)	The United States	1508	13.9 (1.4)	47.00%	*FFQ (Food Frequency Questionnaire)		Intake of energy-dense foods (e.g. cookies and French fries).	Kandel’s depressive symptoms scale for adolescents.	Self-report	Females mean (SD) 11.3 (3.0); males mean (SD) 10.4 (2.9)	Yes
Chi et al. (2020)	China	1794	15.26 (0.46)	56.20%	Nutrition subscale of the Chinese version of the Health Promoting Lifestyle Profile-II (HPLP-II)	HPLP-II scores.		The Chinese version of the 9-item Patient Health Questionnaire (PHQ-9), cut-off 5; Chinese version of the Generalized Anxiety Disorder scale (GAD-7), cut-off 5.	Self-report	The prevalence of depression and anxiety symptoms were 48.2% and 36.7%, respectively.	Depression symptoms only.
Dennison-Farris et al. (2017)	The United States	121	10.5 (1.6)	39.70%	FFQ: Youth Risk Behavior Surveillance survey.	Intake of fruit, salad, potato, carrot and ‘other’ vegetable consumption.		Child Depression Inventory (CDI).	Self-report	Mean (SD) 0.38 (0.29); 12% met criteria for depressive symptoms	No
Farhangi et al. (2018)	Iran	107	17.38 (0.62)	0.00%	*Eating behavioral pattern questionnaire (EBPQ)		Snacking and convenience (frequency of intake of cookies, high-sugar bars and other sweet snacks).	Emotional symptoms subscale of the Strengths and Difficulties Questionnaires (SDQ).	Self-report	The prevalence of emotional disorders (disorder possibly and probable) was 28%	Yes
Ferrer-Cascales et al. (2019)	Spain	527	14.43 (1.52)	45.50%	*Mediterranean Diet Quality Index for children and teenagers (KIDMED).	Mediterranean diet adherence.		Moods and Emotions subscale of the KIDSCREEN-52.	Self-report	Not indicated.	Yes
Fulkerson et al. (2004)	The United States	4734	males 14.9 (1.7); female 14.7 (1.7)	50.21%	*Youth and Adolescent food frequency Questionnaire (YAQ).	Daily servings of fruit and vegetables.	Frequency of eating fast food (days in the past week).	Kandel’s depressive symptoms scale for adolescents	Self-report	Score exceeding 23, *n* = 582; 12%	No
Hayward et al. (2016)	Australia	3295	15.14 (1.12)	54.25%	*Simple Dietary Questionnaire.	Principal components analysis (PCA): healthy diet pattern (strong positive loadings on serves of fruits and vegetables per day).	PCA: unhealthy diet pattern (strong positive loadings for consumption of takeaway foods, sweetened beverages, caffeinated beverages and energy drinks).	Short Moods and Feelings Questionnaire (SMFQ)	Self-report	Mean (SD) 4.82 (5.34); 21.55% exhibited depressive symptoms.	Only for unhealthy eating and depressive symptoms in males.
Hoare et al. (2018)	The United States	3696	15.9 (1.7)	–	Participants completed questionnaires on habitual dietary intake including items asking whether they had eaten fruit or vegetables on the previous day.	Participants were asked ‘How often did you eat fruit or drink fruit juice yesterday?’ with responses ‘didn’t eat’, ‘ate once’ or ‘ate twice or more’. The same item with response options was asked for vegetable consumption.		Center for Epidemiologic Studies Depression Scale (CES-D)	Self-report	21.1% exhibited depression during adolescence.	Fruit consumption in males and females; vegetable consumption in female only.
Huang et al. (2018)	Taiwan	1371	13.6 (SE = 0.01)	51.70%	FFQ and 24-hour dietary recall	Youth Healthy Eating Index-Taiwan (YHEI-TW).		Unhappiness or Depression (UD, 7 items) from the Scale for Assessing Emotional Disturbance (SAED).	Teacher-report	UD scores approx. 7–11	Girls only
Jacka et al. (2010)	Australia	7114	Non-symptomatic 11.6 (0.78); symptomatic 11.6 (0.84)	Non-symptomatic 50.2%; symptomatic 42.7%	14-item dietary questionnaire based on a questionnaire used in the Amherst Health and Activity Study Adult Survey of Child Health Habits, modified to include additional questions about the consumption of breakfast, different types of beverages and takeaway food.	Breakfast every day before school; low fat dairy food at least once per day; at least two serves of fruit per day; and at least four serves of vegetables per day.	Intake of hamburgers, hot dogs or sausages; potato crisps or savory snacks; biscuits, doughnuts, cake, pie or chocolate; and sweet drinks such as soft drinks, cordial, Big M, flavored mineral water, etc.	SMFQ, cut-off 8 for depressive symptoms.	Self-report	SMFQ Symptomatic 33.3%; median SMFQ score was 5 (IQR 2–10).	Yes
Khayyatzadeh et al. (2019)	Iran	750	14.5 (1.5)	0.00%	*147-item FFQ; administered by trained interviewers to estimate.	PCA: Healthy dietary pattern; high intakes of legumes and other vegetables, fish, eggs, yogurt, both cruciferous and green leafy vegetables, tomatoes, garlic, fruits, olives, mayonnaise, both low- and high-fat dairy products.	PCA: Unhealthy dietary pattern; high intakes of refined grains, snacks, red meats, poultry, fish, organ meats, pizza, fruit juices, industrial juice and compote, mayonnaise, nuts, soft drinks, sweets and desserts, coffee and pickle.	Persian version of the Beck Depression Inventory (BDI).	Self-report (administered by trained interviewers)	More than 29% (*n* = 195) of adolescents had mild to severe depression symptoms.	Healthy dietary pattern only.
Kim et al. (2015)	Korea	733	15 (1.5)	0%	*63-item FFQ published by the Korean Health and Nutrition Examination Survey.	Intake of fruits and green vegetables.	Intake of instant foods including ramen, hamburger, pizza, fried foods	Korean version of the BDI, cut-off 16.	Self-report	13.6% exhibited depressive symptoms; cases mean (SD) 21.1 (5.2), controls mean (SD) 5.8 (4.3)	Green vegetable intake and instant foods
Kohlboeck et al. (2012)	Germany	3361	11.15 (0.5)	51%	*82-item FFQ to assess food intake over the last year; collapsed into 11 food categories using the Codex General Standard for Food Additives food category system of the Codex Alimentarius Commission of the Food and Agriculture Organization of the United Nations/World Health Organization.	OMD, a quantitative preventive dietary concept which takes German meal patterns into account; lower in fat and saturated fatty acids and contains ample amounts of plant foods.		Emotional symptoms subscale of the SDQ; cut-off 4 for emotional symptoms.	Parent-report	Males 16.5% exhibited emotional symptoms; females 17.2%	Yes
Kulkarni et al. (2015)	New Zealand	4249	15.2	47.50%	Dietary intake was assessed by using a questionnaire designed for the OPIC study.	Eating breakfast, mid-morning snack and lunch; eating breakfast, mid-morning snack and lunch at home; eating fruits and vegetables; and eating dinner as a family.	Consuming soft drinks, takeaways, unhealthy snacks, fried or high-fat foods, sweet foods.	Emotional functioning subscale of the Pediatric Quality of Life Inventory (PedsQL).	Self-report	Mean score 75.6	Yes
Oellingrath et al. (2014)	Norway	789	12–13	50%	*FFQ assessing habitual daily consumption of 40 food items.	PCA, Varied Norwegian pattern: unrefined plant foods, fish, water and regular breakfast and lunch.	PCA, junk/convenient pattern: high-energy processed fast foods, refined grains, cakes and sweets.	Emotional symptoms subscale of the SDQ.	Parent-report	2% prevalence of possible/likely emotional disorder.	No
Puloka et al. (2017)	New Zealand	8500	13–17	51.30%	Survey covering a range of areas relevant to the health and well-being of adolescents.	Intake of fruit, vegetable, sandwich, milk, breakfast, lunch, dinner, and home as a source of lunch and eating together as a family.	Intake of takeaway food, foods from dairies or petrol stations, chocolate, sweets or lollies, potato, chips, burger rings, twisties and other crisps, meat pies or sausages, fizzy or soft drinks and energy drinks.	Reynold’s Adolescent Depression Scale, cut-off 28 indicating significant depressive symptoms.	Self-report	Significant depressive symptoms in 30% of adolescents in the lowest quartile of healthy eating and 15% in the highest quartile for unhealthy eating.	Yes
Renzaho et al. (2011)	Australia	3370	38% ages 4–7, 48% ages 8–11 and 14% age 12.	51.80%	FFQ	Servings of fruits and vegetables usually eaten per day.		Emotional symptoms subscale of the SDQ.	Parent-report	Not indicated.	Fruit consumption only in boys; fruit and vegetable consumption in girls.
Robinson et al. (2011)	Australia	1784	14.01 (0.2)	51.00%	*Commonwealth Scientific and Industrial Research Organization (CSIRO) FFQ.	Fruit and vegetable intake, servings per day.	Extra food servings per day, including meat pies, hot chips, pizza, fried food, cakes, chocolate, biscuits, mayonnaise, dressings, soft drinks, ice cream.	Internalizing problems subscale of the Parent report Child Behaviour Checklist for Ages 4–18 (CBCL).	Parent-report	13% showing scores above the clinical cut-point for internalizing problems	Extra foods only
Sakai et al. (2017)	Japan	3963	18	0.00%	*152-item diet history questionnaire (DHQ).	Higher intake of dairy products, fruit, seaweed, soya products, vegetables, EPA + DHA, dietary fiber, Ca, Mg, Fe, folate and vitamin C and lower intakes of confectioneries, sugar, soft drinks and total and saturated fats.		CES-D, cut-off 23	Self-report	22% of young women exhibited depressive symptoms.	Yes
Shivappa et al. (2018)	Iran	300	Normal symptoms 16.2 (1); mild depressive symptoms 16.3 (1.1)	0%	*168-item FFQ; collected by specifically trained professional interviewers through private face-to-face interviews.		DII, pro-inflammatory components and anti-inflammatory components. Higher scores indicate higher pro-inflammatory diet.	The Persian version of Depression, Anxiety, Stress Scale-21 (DASS-21); cut-off 9 for mild levels of depressive symptoms.	Self-report	14.33% exhibited mild depressive symptoms.	Yes
Sinclair et al. (2016)	Fiji	7237	15.6 (1.4) baseline; 17.4 (0.9) follow-up	47.40% baseline; 44.2% follow-up	Adolescent Behaviours, Attitudes and Knowledge Questionnaire (ABAKQ).	Availability of fruit at home; daily servings of fruit; daily servings of vegetables; eating fruit after school; and consuming cordial/fruit drinks in the last 5 school days.	Takeaway foods for dinner; non-diet soft drinks in the last 5 school days; chocolates/sweets after school; pies/fried foods/takeaways after school; snack foods after school; purchasing snack foods after school in the last 5 school days; purchasing meals from a takeaway shop; availability at home of snack foods, chocolates/sweets, non-diet soft drinks.	Emotional functioning subscale of the PedsQL.	Self-report	Baseline mean score 64.1 (17.6); follow-up mean score 64.4 (16.3).	Healthy eating only
Tanaka and Hashimoto (2019)	Japan	858	Junior high school students 13.98 (0.86) and senior high school students 17.09 (0.88)	43%	FFQ; Food groups in the Japanese food guide spinning top.	Intake of green and yellow vegetables and fruit.		Japanese version of the CES-D, cut-off 16.		Mean (SD) in junior high school boys 12.15 (7.88) and girls 12.57 (7.12); mean (SD) in senior high school boys 21.28 (10.63) and girls 21.88 (10.83).	Green and yellow vegetable intake only.
Tehrani et al. (2018)	Iran	263	16.2 (0.97)	0	*168-item FFQ.	MSDPS; 13-items according to the Mediterranean diet pyramid; only 12 scores were obtained because the participants’ religious beliefs prohibited them responding the frequency of alcoholic beverages consumption.		The Persian version of DASS-21; cut off 10.	Not indicated, presuming self-report since average age is ~16.	The mean depression, anxiety and stress scores were 9.89, 8.43 and 14.00, respectively.	Depression and healthy eating only (not anxiety)
Thomas et al. (2020)	Australia	2644	1367 ages 5–10; 1277 ages 11–15	50% ages 5–10; 48.9% ages 11–15	Parent estimates of servings of fruit and vegetables per day and frequency of discretionary food intake.	Fruit and vegetable intake.	Intake of processed meat, salty snacks, fried potato products, sugary baked goods, fast food, cordial or other sugar-sweetened beverages and confectionary.	Emotional symptoms subscale of the SDQ.	Parent-report	The prevalence of emotional symptoms was 10.5% in the 5–10 age group and 15.5% in the 11–15 age group.	No
Weng et al. (2012)	China	5003	13.21 (0.99)	52.09%	38-item FFQ.	Gruel, oatmeal, whole grains, fresh yellow or red vegetables, fruit and soya milk.	Preserved fruit, a sweet course, frozen confection, yogurt, chocolate, candy and carbonated drinks.	Chinese version of the Depression Self-rating Scale for Children (DSRS), cut-off 15; Chinese version of the Screen Scale for Child Anxiety Related Emotional Disorders (SCARED), cut-off 23.	Self-report	The prevalence of depression symptoms, anxiety disorders and the coexistence of both were 11.2%, 14.6% and 12.6%, respectively.	Pure depression and healthy eating, pure depression and pure anxiety and unhealthy.
Xu et al. (2019)	China	14,500	14.9 (1.8)	50.70%	SQ-FFQ.	Intake of eggs, fruit, vegetables, pure milk.	Takeaway foods by box and plastic bag, hot soy milk with plastic straw and plastic cup with hot porridge.	Emotional, problems subscale of the Multi-dimensional Sub-health Questionnaire of Adolescents (MSQA)	Self-report	27.6% exhibited emotional problems	Yes
Xu et al. (2020)	China	14,500	14.9 (1.8)	50.70%	FFQ		Fast food (FF) intake including Western-style FFs (e.g. McDonald’s), Chinese FFs (e.g. Shaxian snacks), takeaway FFs (e.g. Meituan takeaways), foods brought from the school cafeteria and those brought from off-campus restaurant packed in a disposable fast food box or plastic bags.	CDI, cut-off 19	Self-report	27.3% exhibited depression symptoms.	Yes.
Yang et al. (2019)	Taiwan	503	17.30 (1.34)	0.00%	Health Promoting Lifestyle Profile (HPLP)	HPLP scores.		CES-D, cut-off 16	Self-report	48.1% exhibited depression symptoms.	Yes
*Prospective*											
Andersen et al. (2013)	Denmark	2181	15 baseline, 18 follow-ups	45.90%	Eating habits at age 15 and 18 years.		Reduction in fruit and vegetable intake between age 15 and 18.	CES-D	Self-report	Score of 4–12 at follow-up at baseline = 10.7% and follow-up = 10.8%	Yes
Aparicio et al. (2017)	Spain	165	13.46 (0.92)	35.76%	*45-item SQ-FFQ.		Sweet and fatty food pattern: sweets, soft drinks, sweet dairy products, baked goods and chocolates, and savory snacks.	CDI, Spanish version, cut-off 17; SCARED, Spanish version, cut-off 32 for more severe anxiety.	Self-report	59.43% girls and 62.71% boys presented some emotional symptoms (note MH measures were collapsed)	Females only for sweet and fatty foods.
Hoare et al. (2020). *Note*: Only cross-sectional results at age 14 were used.	The United Kingdom	9369	7 baseline; 14 follow-up	Not indicated.	Fruit and vegetable consumption were measured using the question ‘How often do you eat at least 2 portions of [fruit/vegetables]?’ with responses never, some days but not all days, and every day.	Fruit and vegetable intake.		SDQ at age 7; SMFQ at age 14, cut-off 8.	Parent-report at age 7, self-report at age 14.	22.8% self-reported mental health problems at age 14.	Yes
Jacka et al. (2011)	Australia	2915 baseline; 1949 follow-up	Majority < 15.	Baseline males 56%; final follow-up males 54%	Nutrition subscale of an 84-question survey using Personal Diary Assistants.	Eating lunch brought from home; consuming two or more fruit serves per day; four or more vegetable serves per day; fruit and/or sandwiches as after-school snacks; generally avoiding biscuits, potato chips, pies, hot chips, fried foods, chocolate, sweets, ice-creams as after-school snacks; and both consuming healthy after-school snacks and avoiding unhealthy after school snacks.	Biscuits, potato chips, other snacks after school; Pies, takeaways or fried foods such as French fries after school; Chocolates, lollies, sweets or ice-creams after school, non-diet soft drinks, soft drinks, fruit drinks and cordials, takeaway food, buy snack food from shop/takeaway foods.	Emotional functioning subscale of the PedsQL.	Self-report	Not indicated.	Healthy eating only
Jacka et al. (2013)	The United Kingdom	2789	11–12 baseline; 13–14 follow-up.	48.80%	**Health and Behaviours of Teenagers Study (HABITS)	Breakfast consumption, fruit and vegetable intake	Fast foods, snacks and biscuits high in saturated fats and sugars.	SMFQ, cut-off 8.	Self-report	24.5% cases on SMFQ	Yes
Jacka et al. (2013)	Norway	23,020	Follow-up at 1.5, 3 and 5 years	Not indicated.	36-item FFQ at 1.5 years and 37-item FFQ at 3 years.	Intake of white fish, oily fish, boiled vegetables, raw vegetables, fruit, bread with fish products, egg, bread with meat, Norwegian brown cheese and fish products.	Intake of chips, buns, cakes, waffles, chocolate, cookies, sweets, soda, ice cream, popsicles, bread with jam or honey, pizza and soda with artificial sweeteners.	Short-form of the CBCL.	Parent-report	Not indicated	Yes
Michels et al. (2018)	Switzerland	291	3.9–6.3 baseline; 4.6–7.1 follow-up	47.00%	*SQ-FFQ	Fruit and vegetable intake.	Fatty food intake.	Emotional functioning subscale of the PedsQL 4.0 for children 5–7 years.	Parent-report	Baseline mean (SD) 72.8 (14.6); follow-up mean (SD) 72.4 (13.4)	No (unadjusted Pearson correlations)
Wiles et al. (2009)	The United Kingdom	4541 (emotional problems)	4.5 baseline; 7 follow-up		Postal FFQ		Intake of high-fat processed foods (burgers, coated poultry) and snack foods high in fat and/or sugar (such as crisps and chocolate), which tend to be of poor nutritional quality.	Emotional symptoms subscale of the SDQ.	Parent-report	38.4% high emotional problems (imputed 24.7% to account for missingness)	No
Wilson et al. (2020)	Australia	1005	12.5 baseline; 33–41 follow-up	51.20%	24-hour food and drink record at baseline	Dietary Guidelines Index (DGI)		Lifetime version of the Composite International Diagnostic Interview (CIDI) at follow-up	Self-report for diet only.	25.4% reported a mood disorder at follow-up.	No
Winpenny et al. (2018)	The United Kingdom	603	14.5 (3.5) baseline; 17.5 (4.1) follow-up.	40.00%	4-day diet diary, including 2 weekdays and 2 weekend days.	Alternative Mediterranean diet score: Intake of vegetables, legumes, fruit, nuts, whole grains, red and processed meat, fish, ratio of monounsaturated to saturated fat, ethanol.		Moods and Feelings Questionnaire (MFQ); cut-off 20	Self-report	Baseline mean (SD) 14.3 (9.7); follow-up mean (SD) 13.2 (9.6)	No
Wu et al. (2018)	Canada	4861	10 or 11 baseline; 18 follow-up.	49.20%	*Diet Quality Index-International score based on student responses to the YAQ and Canadian Nutrient Files.	The Diet Quality Index-International.		ICD-9 and ICD-10 codes for internalizing disorders, physician diagnosis.	Self-report report for diet only.	23.7% diagnosed with an internalizing disorder during the time between completing the survey (at age 10 or 11 years) and turning 18 years old.	No

* indicates the dietary intake measure has undergone validity testing.OPIC: obesity prevention in communities; IQR: inter quartile range.

#### Study quality

Eighteen studies used dietary intake measures that had undergone validity-testing, 12 of which were assessed in child and adolescent age groups, and 6 in adults aged 19 and older. All studies used validated measures of depressive, anxious or broader internalizing symptoms. The average study quality scores were 5.7 out of 7 for cross-sectional studies, 5.8 out of 8 for prospective studies, and 8 out of 8 for the case-control study. Additional details of the quality assessment can be found in Supplementary Tables 1 to 3.

### Meta-analysis

#### Healthy dietary pattern and mental health

##### Internalizing symptoms

**Figure 2.**
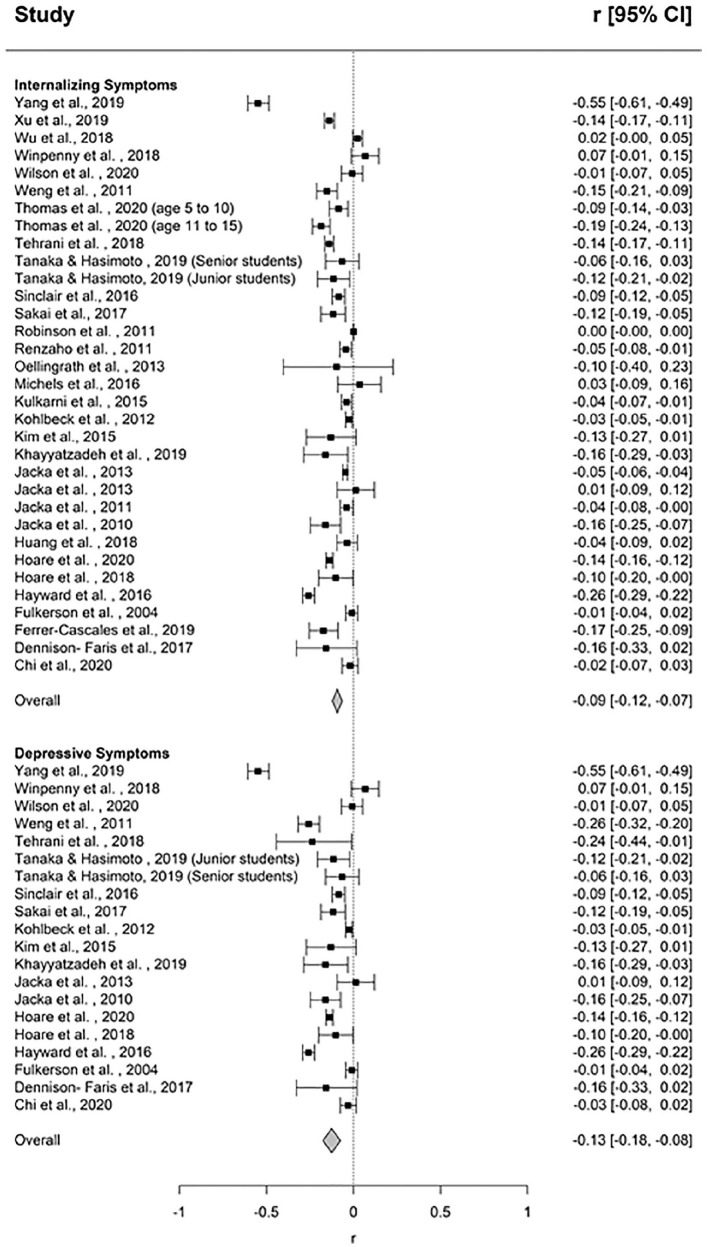
Forest plot of effect sizes for the association between healthy dietary pattern and internalizing and depressive symptoms.

**Figure 3.**
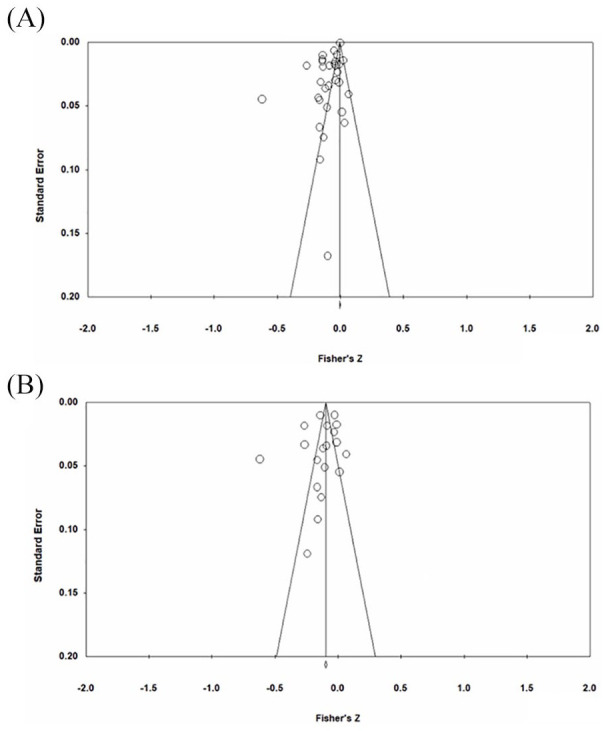
Funnel plot of standard error by Fisher’s *Z*, for effect sizes describing the association between healthy eating and mental health outcomes. (A) Internalizing symptoms and (B) depressive symptoms.

There was a small but significant pooled effect size between healthy dietary patterns and internalizing symptoms (*k* = 31, *r* = –0.09, *p* < 0.000, 95% CI [–0.12, 0.06]) (Figure 2). The funnel plot revealed asymmetry (Figure 3A), and Egger’s test indicated this asymmetry was significant (*p* < 0.001). The adjusted mean effect size using the trim and fill analysis was *r* = –0.12 (95% CI [–0.182, –0.07]). There was significant heterogeneity between studies (*Q* = 992.767, *p* < 0.0001, I^2^ = 96.777). One outlier was identified. The mean correlation following its removal was *r* = –0.08 (95% CI [–0.10, –0.05], *p* < 0.0001) and significant heterogeneity remained (*Q* = 795.646, *p* < 0.0001, *I*^2^ = 96.355). The results of categorical and continuous moderator analyses are presented in Tables 2 and 4, respectively. Of the categorical moderators, study informant and study design were significant. Larger effect sizes were observed for studies using self-report measures (*k* = 23, *r* = –0.11, 95% CI [–0.14, –0.08], *p* < 0.0001) compared to parent or teacher-report (*k* = 8, *r* = –0.04, 95% CI [–0.09, 0.01], *p* = 0.10) (*Q* = 5.09, *p* = 0.02). Larger effect sizes were observed for cross-sectional studies (*k* = 22, *r* = –0.12, 95% CI [–0.16, –0.09], *p* < 0.0001) compared to prospective studies (*k* = 9, *r* = –0.03, 95% CI [–0.09, 0.03], *p* = 0.39) (*Q* = 7.33, *p* = 0.01). Percent male participants was the only significant continuous moderator (*k* = 28, *b* = 0.004, 95% CI [0.00, 0.01], *p* < 0.0001) such that as the percentage of males in samples increased, so too did effect sizes.

**Table 2.** Categorical moderator analyses for the association between healthy dietary pattern and internalizing symptoms.

Moderator	*k*	Correlation	Lower limit	Upper limit	*Q*	*p*
*Depression, anxiety and emotional symptoms*						
Effect size						
Not pooled	19.00	–0.10	–0.14	–0.07	0.82	0.37
Pooled	12.00	–0.08	–0.12	–0.03		
Study design						
Cross-sectional	22.00	–0.12	–0.16	–0.08		
Prospective	9.00	–0.03	–0.09	0.03	7.33	0.01
Informant, diet						
Self-report	23.00	–0.11	–0.14	–0.08	5.09	0.02
Parent-report	8.00	–0.04	–0.09	0.01		
Diet measure						
Not validated	15.00	–0.09	–0.13	–0.05	0.09	0.77
Validated	16.00	–0.10	–0.14	–0.06		
*Depressive symptoms*						
Effect size						
Not pooled	12	–0.15	–0.22	–0.08	0.80	0.37
Pooled	7	–0.10	–0.19	–0.01		
Study design						
Cross-sectional	15	–0.16	–0.22	–0.10	5.63	0.02
Prospective	4	–0.01	–0.12	0.11		
Informant, diet						
Self-report	1	–0.03	–0.25	0.20	0.85	0.36
Parent-report	18	–0.14	–0.19	–0.08		
Diet measure						
Not validated	8	–0.11	–0.19	–0.02	0.49	0.48
Validated	11	–0.15	–0.22	–0.07		

Significance codes: *p < 0.05; **p < 0.01; ***p ⩽ 0.001.

**Table 3.** Categorical moderator analyses for the association between unhealthy dietary pattern and internalizing symptoms.

Moderator	*k*	Correlation	Lower limit	Upper limit	*Q*	*p*
*Depression, anxiety and emotional symptoms*						
Effect size						
Not pooled	19	0.114	0.066	0.162	0.989	0.32
Pooled	6	0.065	–0.02	0.149		
Study design						
Cross-sectional	16	0.119	0.059	0.177	0.669	0.413
Prospective	9	0.078	0.001	0.155		
Informant, diet						
Self-report	**18**	**0.14** ***	**0.08**	**0.20**	**5.67**	**0.02**
Parent or teacher-report	**7**	**0.01**	**–0.09**	**0.10**		
Diet measure						
Not validated	12	0.125	0.063	0.186	1.008	0.315
Validated	13	0.078	0.012	0.144		
*Depressive symptoms*						
Effect size						
Not pooled	10	0.099***	0.042	0.154	0.343	0.558
Pooled	3	0.131**	0.038	0.222		
Study design						
Cross-sectional	10	0.12**	0.042	0.196	0.886	0.347
Prospective	3	0.072*	0.008	0.135		
Informant, diet						
Self-report	12	0.12**	0.035	0.203	0.355	0.551
Parent-report	1	0.031	–0.244	0.302		
Diet measure						
Not validated	5	0.105	–0.015	0.223	0.017	0.897
Validated	8	0.116*	0.015	0.214		

Significance codes: *p < 0.05; **p < 0.01; ***p ⩽ 0.001.

Bolded values indicate significance of p < 0.05.

**Table 4.** Continuous moderator analyses for the association between healthy dietary pattern and internalizing symptoms.

Moderator	*k*	Correlation	SE	Lower limit	Upper limit	*p* value
*Depression, anxiety and emotional symptoms*						
Percent boys	28	0.003***	0.00	0.00	0.01	0.00
Age	31	–0.01	0.01	–0.02	0.00	0.14
Study quality	31	–0.15	0.12	–0.39	0.09	0.22
Percent high SES	18	0.00	0.00	0.00	0.00	0.19
Percent internalizing symptoms	15	0.00	0.00	0.00	0.00	0.22
Percent overweight and obese	7	0.00	0.00	–0.01	0.00	0.44
Year	31	0.00	0.00	–0.01	0.00	0.07
*Depressive symptoms*						
Percent boys	17	0.00**	0.00	0.00	0.01	0.03
Age	19	–0.01	0.01	–0.04	0.01	0.37
Study quality	19	–0.29	0.27	–0.81	0.23	0.28
Percent high SES	9	0.00	0.00	0.00	0.01	0.16
Percent depressive symptoms	15	0.00	0.00	0.00	0.00	0.31
Percent overweight and obese	4	0.00	0.00	–0.01	0.00	0.36
Year	19	0.00	0.01	–0.01	0.01	0.39

SES: socioeconomic status; BMI: body-mass index (kg/m2).

Significance codes ***p < .001; **p < .01.

##### Depressive symptoms

There was a small, significant pooled association between healthy dietary patterns and depressive symptoms (*k* = 19, *r* = –0.13, *p* < 0.001, 95% CI [–0.18, –0.08]) (Figure 2). The funnel plot did not reveal asymmetry (Figure 3B), and Egger’s test was non-significant (*p* = 0.37). There was significant heterogeneity between studies (*Q* = 375.775, *p* < 0.0001, *I*^2^ = 94.944). One outlier was identified. The mean correlation following its removal was *r* = –0.10 (95% CI [–0.14, –0.06], *p* < 0.0001) and significant heterogeneity remained (*Q* = 237.424, *p* < 0.0001, *I*^2^ = 92.84). Study design was a significant moderator. Larger effect sizes were observed for cross-sectional studies (*k* = 15, *r* = –0.16, 95% CI [–0.22, –0.10], *p* < 0.0001) compared to prospective studies (*k* = 4, *r* = –0.01, 95% CI [–0.12, 0.11], *p* = 0.92), which was non-significant. Percent male participants was also a significant moderator (*k* = 17, *b* = 0.003, 95% CI [0.00, 0.01], *p* = 0.04), with effect sizes increasing as the percentage of males in samples increased.

#### Unhealthy dietary pattern and mental health

##### Internalizing symptoms

**Figure 4.**
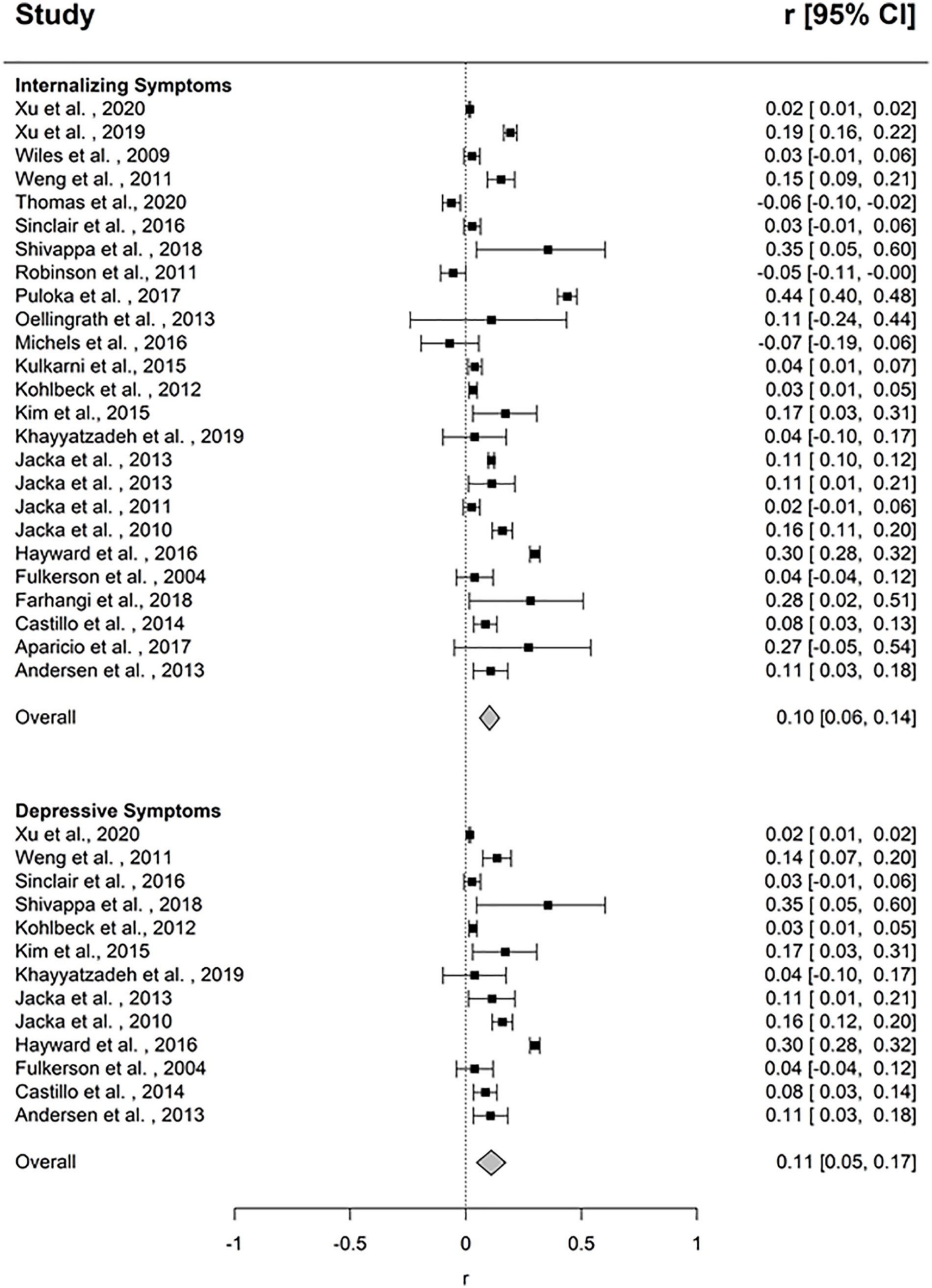
Forest plot of effect sizes for the association between unhealthy dietary pattern and internalizing and depressive symptoms.

**Figure 5.**
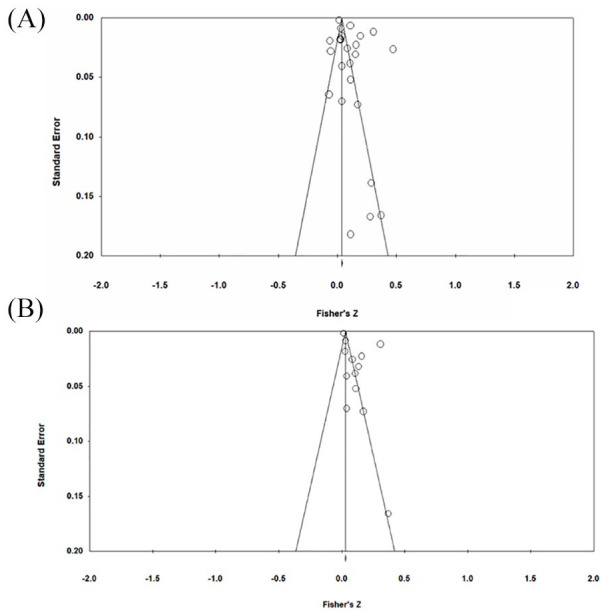
Funnel plot of standard error by Fisher’s *Z*, for effect sizes describing the association between unhealthy eating and symptoms mental health outcomes. (A) Internalizing symptoms and (B) depressive symptoms.

There was a small but significant association between unhealthy dietary pattern and symptoms of depression, anxiety and emotional problems (*k* = 25, *r* = 0.10, *p* < 0.001, 95% CI [0.06, 0.14]), suggesting that symptoms of depression and anxiety are positively correlated with unhealthy eating (Figure 4). The funnel plot revealed asymmetry (Figure 5A), and Egger’s test indicated this asymmetry was significant (*p* = 0.05); however, the trim and fill analysis did not identify any studies to be removed. There was significant heterogeneity between studies (*Q* = 1206.283, *p* < 0.0001, *I*^2^ = 97.928). One outlier was identified. The mean correlation following its removal was *r* = 0.08 (95% CI [0.04, 0.12], *p* < 0.0001), and significant heterogeneity remained (*Q* = 929.868, *p* < 0.0001, *I*^2^ = 98.316). Results of categorical and continuous moderators are presented in Tables 3 and 5, respectively. Study informant was a significant moderator, with larger effect sizes reported for self-report measures (*k* = 18, *r* = 0.14, 95% CI [0.08, 0.20], *p* < 0.001) compared to parent-report measures (*k* = 7, *r* = 0.01, 95% CI [–0.09, 0.10], *p* = 0.91) (*Q* = 5.67, *p* = 0.02). Percent overweight and obese participants was also a significant moderator, with stronger effect sizes associated with a higher percentage of overweight and obesity in the sample (*k* = 4, *b* = 0.03, 95% CI [0.01, 0.06], *p* = 0.02).

**Table 5.** Continuous moderator analyses for the association between unhealthy dietary pattern and internalizing symptoms.

Moderator	*k*	Correlation	SE	Lower limit	Upper limit	*p* value
*Depression, anxiety and emotional symptoms*						
Percent boys	22	0.00	0.00	0.00	0.00	0.38
Age	25	0.01	0.01	0.00	0.03	0.11
Study quality	25	0.11	0.16	–0.20	0.41	0.50
Percent high SES	13	0.00	0.00	–0.01	0.00	0.57
Percent internalizing symptoms	12	0.00	0.00	0.00	0.00	0.78
Percent overweight and obese	4	0.03	0.01	0.01	0.06	0.02
Year	25	0.00	0.01	–0.01	0.01	0.41
*Depressive symptoms*						
Percent boys	12	0.00	0.00	0.00	0.00	0.67
Age	13	0.00	0.02	–0.04	0.04	0.88
Study quality	13	0.17	0.34	–0.51	0.84	0.63
Percent high SES	8	0.00	0.00	–0.01	0.01	0.89
Percent depressive symptoms	10	0.00	0.00	0.00	0.00	0.90
Percent overweight and obese	–	–	–	–	–	–
Year	13	0.00	0.01	–0.02	0.02	0.97

SES: socioeconomic status; BMI: body-mass index (kg/m^2^).

##### Depressive symptoms

There was a small but significant association between unhealthy dietary pattern and depressive symptoms (*k* = 13, *r* = 0.11, *p* = 0.001, 95% CI [0.05, 0.17]), suggesting that symptoms of depression are positively correlated with unhealthy eating (Figure 4). The funnel plot revealed asymmetry (Figure 5B); however, Egger’s test indicated this asymmetry was non-significant (*p* = 0.10). There was significant heterogeneity between studies (*Q* = 657.387, *p* < 0.0001, I^2^ = 98.175). One outlier was identified. The mean correlation following its removal was *r* = 0.11 (95% CI [0.04, 0.17], *p* = 0.002) and significant heterogeneity remained (*Q* = 653.126, *p* < 0.0001). No significant moderators were identified.

## Discussion

This meta-analysis found that healthy dietary pattern was significantly associated with fewer internalizing symptoms and particularly depressive symptoms. In contrast, unhealthy dietary pattern was significantly associated with greater symptoms of depression and internalizing symptoms more broadly. All pooled effect sizes are considered small in magnitude and are comparable in magnitude, suggesting both healthy and unhealthy dietary patterns are meaningfully associated with internalizing and depressive symptoms.

The association between depressive symptoms and dietary patterns may be a result of several potential mechanisms, including biological links such as inflammation, gut-brain axis and BDNF, as well as psychosocial explanations. One leading theory that may link diet and mental health symptoms, particularly depressive symptoms, is that of inflammation (Wang et al., 2019), as unhealthy eating may perpetuate a pro-inflammatory state (Perez-Cornago et al., 2014). Among adults, a recent meta-analysis found that a pro-inflammatory diet was associated with an increased risk of depression, especially in women (Wang et al., 2019). Similarly, another recent meta-analysis found that pro-inflammatory cytokines are increased among adolescents with depression and internalizing disorders (Belem da Silva et al., 2017; Colasanto et al., 2020). Although no intervention studies exist in the child and youth populations, lower rates of inflammation after a change in diet were associated with fewer depressive symptoms in one study in adults (Perez-Cornago et al., 2014). Also, diet is also a central factor in how the gut microbiome is configured, with a growing body of literature now demonstrating the association between gut microbiota and mental health disorders (Cryan and Dinan, 2012). A third potential mechanism of association involves a neurochemical, BDNF. BDNF is critical to neuronal functions such as growth and differentiation, and is an important factor in neuroplasticity (Lachance and Ramsey, 2015) with hippocampal levels shown to decrease in response to a high-fat, refined sugar diet in animal models (Jacka and Berk, 2007). In terms of psychosocial explanations, it is possible that under periods of stress, individuals are more likely to choose more palatable (high-sugar, high-fat, processed) foods in an effort to self-soothe (Singh, 2014). If and when this becomes a chronic behavior, overconsumption of calorie-dense foods may contribute to depression through biological and psychosocial pathways (Singh, 2014). Finally, another psychosocial pathway to consider would be the impact of food insecurity in a family, which has been found to be predictive of higher levels of children’s mental health conditions (Melchior et al., 2012). Thus, there are several psychosocial variables with respect to the relationship between eating and mental health that should be considered, including poverty and food insecurity, availability of parents and families eating together, and the family culture around the enjoyment of food.

In the current meta-analysis, participant sex was a significant moderator in studies of healthy dietary patterns and internalizing symptoms, with studies with higher percentages of male participants having stronger effect sizes. In one of the few prospective studies available, Trapp et al. (2016) observed that females consuming a Western dietary pattern at 14 years were more likely to have externalizing behaviors within clinical threshold at 17 years (Trapp et al., 2016). Although these findings were not aligned with those of the current meta-analysis, it suggests that there may be a need to consider possible sex differences in future research. In addition, participant body weight (overweight or obese) was also a significant moderator, with higher percentages of overweight and obese participants associated with stronger effect sizes in studies of unhealthy dietary patterns and symptoms of internalizing problems. Studies of internalizing symptoms and weight are mixed. Prospective studies have found internalizing symptoms to be associated with later weight gain (Camfferman et al., 2016), and depression in youth associated with higher body mass index (BMI) in adulthood (Korczak et al., 2014). However, a cross-sectional study found no association between emotional problems and weight status among preschool children (Mackenbach et al., 2012). Taken together with the findings in the current study, research suggests the association between body weight and mental health is likely bidirectional, with greater evidence for mental health status influencing later weight gain.

In the current meta-analysis, associations were stronger for studies using child- and adolescent-report, rather than parent- or teacher-report, of dietary intake, for studies of internalizing symptoms and healthy and unhealthy dietary patterns. Informant discrepancy between parents and children has been documented in studies of dietary habits, with one study finding that compared to parents, children tended to report greater allowance of soft drinks and fruit juice, less encouragement to eat breakfast and more availability of soft drinks at home (Rebholz et al., 2014). Children and adolescents may be more aware of their own intake and thus more accurate in reporting on what they eat. Parents may not know what types of foods children consume outside of supervision (e.g. at school). It is also possible that children and adolescents are more willing than their parents to report on certain dietary behaviors, such as the amount and frequency of junk food consumption, as parent responses may be influenced by social desirability (Karver, 2006).

Associations were also stronger for cross-sectional versus longitudinal studies examining depression and/or internalizing symptoms and healthy dietary pattern, with non-significant associations between healthy dietary pattern and future depression and/or internalizing symptoms. In the current study, the pooled association between healthy dietary patterns and depressive symptoms was larger than that of healthy dietary patterns and depressive and/or internalizing symptoms, suggesting the relationship between diet and mental health to be stronger for depression than other forms of internalizing symptoms. Although there is evidence in adult populations that improvements to dietary pattern can improve symptoms of depression (Firth et al., 2019), the reverse may also be true, such that children and adolescents with depression are less likely to engage in healthy eating behaviors. Concurrent associations may be more robust given the tendency for changes in appetite or attitudes toward foods to occur in conjunction with mood difficulties. Indeed, emotional eating—eating in an effort to cope with negative emotions—has been associated with depressive symptoms in adolescent populations (Hou et al., 2013). Furthermore, associations between depression and eating disorder symptoms (e.g. eating large amounts of food in the absence of physical hunger) have been shown to increase in magnitude during adolescence, suggesting that depression and disordered eating behaviors may develop concurrently during youth (Abbasalizad Farhangi et al., 2018). The directionality of associations therefore warrants greater exploration in future research given its implications for targets of intervention.

Dietary pattern may be an important modifiable risk factor for children and youth with respect to symptoms of anxiety and depression. Clinical best-practice guidelines outline the importance of a healthy eating pattern to improve depression in children and youth (National Institute for Health and Care Excellence, 2018). This line of research could help inform clinicians working with youth with depression and other internalizing problems to consider a referral to a dietician. In addition, these findings would also support the need for dieticians to be trained in working with children and adolescents with depression and other internalizing problems. Indeed, other primary care and children mental health clinicians may similarly benefit from dietary education and/or collaboration with dieticians, so that mood and diet concerns can be addressed concurrently.

### Limitations

Several limitations should be considered. First, only one study included in the meta-analysis used physician diagnosis of an internalizing disorder to measure mental health, the remaining used self-report or parent-report measures. Moreover, the majority of studies were of community populations, limiting the generalizability of our findings to clinical samples. Future research should focus on associations between dietary patterns and mood in clinical samples to add to our understanding of this association in the most severely affected and vulnerable populations, as well as circumvent issues of reporting and/or recall bias that may be increased in self-report measures of mental health. Similarly, the majority of studies used food frequency questionnaires. Future studies may achieve greater accuracy of dietary intake through use of more comprehensive measures, such as online recalls. As well, typically the methods used to characterize dietary patterns are not able to account for potential synergies in intake but rather examine the way in which intake of specific foods cluster together or align with an a priori notion of healthy eating, based on an index. Second, only two included studies examined anxiety and dietary patterns, establishing a need for further research on diet and anxiety independent of depression in adolescent populations. Also, it should be considered that the ability to eat in a way which could be characterized as ‘healthy’ may also indicate other measures of health, impacting emotional functioning, such as home environment. Although several moderators were explored, studies were inconsistent in reporting variables including socioeconomic status, body-mass index and exposure to stress as measured through markers of inflammation or physiological mechanisms, reducing the number of studies that could be included in moderator analyses examining these potentially important factors overall. The inclusion of these variables in future research would be important in further defining the relationship between dietary pattern and internalizing symptoms. Finally, as noted in Tables 4 and 5, age was not found to be a continuous moderator. There were limited studies in the early to middle groups. This finding illustrates the need for research among pre-adolescent-aged children.

## Conclusion

This meta-analysis finds that depression and internalizing symptoms are positively associated with unhealthy dietary pattern, and negatively associated with healthy dietary pattern among children and adolescents. The study also highlighted several important moderators. The dietary informant, participant sex and weight status were found to be important variables and may be considered for future research in this area, as well as potential targets for intervention. Future research is also required to establish the direction and mechanism of the association between diet and depression among children and adolescents and to identify the role of dietary improvements in the prevention and treatment of these disorders, in order to impact both mental and physical health outcomes.

## Supplemental Material

sj-docx-1-anp-10.1177_00048674211031486 – Supplemental material for Dietary patterns and internalizing symptoms in children and adolescents: A meta-analysis Click here for additional data file. Supplemental material, sj-docx-1-anp-10.1177_00048674211031486 for Dietary patterns and internalizing symptoms in children and adolescents: A meta-analysis by Laura Orlando, Katarina A Savel, Sheri Madigan, Marlena Colasanto and Daphne J Korczak in Australian & New Zealand Journal of Psychiatry
