# Supplementary material for: Dietary patterns and internalizing symptoms in children and adolescents: A meta-analysis
Source: Aust N Z J Psychiatry. 2021 Jul 27;56(6):617–41. doi: 10.1177/00048674211031486 (PMC9131419; doi:10.1177/00048674211031486)
Supplement: sj-docx-1-anp-10.1177_00048674211031486 – Supplemental material for Dietary patterns and internalizing symptoms in children and adolescents: A meta-analysis [file sj-docx-1-anp-10.1177_00048674211031486.docx]

**Supplemental Information/ Appendix**

**Search Criteria:**

(“child*”, or “youth*”, or “teen*”, or “adolesc*”, or “young*”, or “infan*”, or “baby” or “pedia*”) AND (“depress*” or “depressive symptom*” or “anxiety*” or “internali” or “mood*” or “affective disorder*” or “emotion*”) AND (“diet*” or “nutrit*” or “energy intake*” or “calor*” or “kcal” or “serving size” or “portion size”)

**MEDLINE Search String**

1. exp diet/,

2. (diet* or energy intake* or caloric intake* or portion size* or serving size* or calorie* or cal or kcal or caloric restriction or calorie restriction or food restriction or caloric intake restriction or fasting or calorie intake* or food portion*).tw,kf.,

3. exp nutritional requirements/ or nutritional status/,

4. (nutrition or nutritional or daily recommended allowance* or daily recommended intake* or reference daily intake* or food guide pyramid).tw,kf.,

5. or/1-4,

6. affective symptoms/ or depression/,

7. mood disorders/ or depressive disorder/ or depressive disorder, major/ or depressive disorder, treatment-resistant/ or dysthymic disorder/ or seasonal affective disorder/ or cyclothymic disorder/,

8. (depression or depressions or depressive or affective symptom* or affective disorder* or alexithymia or alexithymias or emotional disturbance* or emotional disorder* or emotional dysfunction or emotional illness or affective neurosis or melancholia or melancholias or mood disorder* or mood disturbance* or paraphrenia or paraphrenias or psychoses or psychosis or dysthymia or dysthymic disoder* or mental fatigue or cyclothymic disorder* or cyclothymic personalit* or cyclophrenia or cyclothymia).tw,kf.,

9. anxiety/ or anxiety disorders/,

10. (worry or apprehension or angst or anxiousness or anxiety or anxieties or fear or fears or hypervigilance or nervousness).tw,kf.,

11. or/6-10,

12. exp child/ or exp infant/ or pediatrics/,

13. (infan* or newborn* or "new born*" or perinat* or neonat* or baby or baby* or babies or toddler* or minors* or boy or boys or boyfriend or boyhood or girl* or kid or kids or child* or schoolchild* or adolescen* or juvenil* or youth* or teen* or pre-pubesc* or prepubesc* or "under* age*" or pubescen* or pediatric* or paediatric* or peadiatric* or prematur* or pre-term or preterm*).mp. or (child* or adolesc* or pediat* or paediat*).jn.,

14. 12 or 13,

15. 5 and 11 and 14

**EMBASE Search String**

1. exp diet/

2. caloric intake/ or portion size/ or caloric restriction/ or diet restriction/

3. (diet* or energy intake* or caloric intake* or portion size* or serving size* or calorie* or cal or kcal or caloric restriction or calorie restriction or food restriction or caloric intake restriction or fasting or calorie intake* or food portion*).tw,kw.

4. exp nutritional requirement/ or nutritional status/

5. (nutrition or nutritional or daily recommended allowance* or daily recommended intake* or reference daily intake* or food guide pyramid).tw,kw.

6. or/1-5

7. depression/ or major depression/ or treatment resistant depression/

8. emotional disorder/ or mood disorder/ or seasonal affective disorder/ or dysthymia/ or cyclothymia/

9. (depression or depressions or depressive or affective symptom* or affective disorder* or alexithymia or alexithymias or emotional disturbance* or emotional disorder* or emotional dysfunction or emotional illness or affective neurosis or melancholia or melancholias or mood disorder* or mood disturbance* or paraphrenia or paraphrenias or psychoses or psychosis or dysthymia or dysthymic disoder* or mental fatigue or cyclothymic disorder* or cyclothymic personalit* or cyclophrenia or cyclothymia).tw,kw.

10. anxiety/ or anxiety disorder/

11. (worry or apprehension or angst or anxiousness or anxiety or anxieties or fear or fears or hypervigilance or nervousness).tw,kw.

12. or/7-11

13. exp child/ or exp infant/ or pediatrics/

14. (infan* or newborn* or "new born*" or perinat* or neonat* or baby or baby* or babies or toddler* or minors* or boy or boys or boyfriend or boyhood or girl* or kid or kids or child* or schoolchild* or adolescen* or juvenil* or youth* or teen* or pre-pubesc* or prepubesc* or "under* age*" or pubescen* or pediatric* or paediatric* or peadiatric* or prematur* or pre-term or preterm*).mp. or (child* or adolesc* or pediat* or paediat*).jn.

15. 13 or 14

16. 6 and 12 and 15

**PsychINFO Search String**

1. diets/

2. (diet* or energy intake* or caloric intake* or portion size* or serving size* or calorie* or cal or kcal or caloric restriction or calorie restriction or food restriction or caloric intake restriction or fasting or calorie intake* or food portion*).tw,kw.

3. dietary restraint/ or food intake/ or nutrition/

4. (nutrition or nutritional or daily recommended allowance* or daily recommended intake* or reference daily intake* or food guide pyramid).tw,kw.

5. or/1-4

6. affective disorders/ or major depression/ or dysthymic disorder/

7. (depression or depressions or depressive or affective symptom* or affective disorder* or alexithymia or alexithymias or emotional disturbance* or emotional disorder* or emotional dysfunction or emotional illness or affective neurosis or melancholia or melancholias or mood disorder* or mood disturbance* or paraphrenia or paraphrenias or psychoses or psychosis or dysthymia or dysthymic disoder* or mental fatigue or cyclothymic disorder* or cyclothymic personalit* or cyclophrenia or cyclothymia).tw,kw.

8. anxiety/ or anxiety disorders/

9. (worry or apprehension or angst or anxiousness or anxiety or anxieties or fear or fears or hypervigilance or nervousness).tw,kw.

10. or/6-9

11. exp child/ or exp infant/ or pediatrics/

12. (infan* or newborn* or "new born*" or perinat* or neonat* or baby or baby* or babies or toddler* or minors* or boy or boys or boyfriend or boyhood or girl* or kid or kids or child* or schoolchild* or adolescen* or juvenil* or youth* or teen* or pre-pubesc* or prepubesc* or "under* age*" or pubescen* or pediatric* or paediatric* or peadiatric* or prematur* or pre-term or preterm*).mp. or (child* or adolesc* or pediat* or paediat*).jn.

13. 11 or 12

14. 5 and 10 and 13

**Web of Science Search String**

1. TS=(diet* or "energy intake*" or "caloric intake*" or "portion size*" or "serving size*" or calorie* or cal or kcal or "caloric restriction" or "calorie restriction" or "food restriction" or "caloric intake restriction" or fasting or "calorie intake*" or "food portion*" or nutrition or nutritional or "daily recommended allowance*" or "daily recommended intake*" or "reference daily intake*" or "food guide pyramid")
2. TS=(depression or depressions or depressive or "affective symptom*" or "affective disorder*" or alexithymia or alexithymias or "emotional disturbance*" or "emotional disorder*" or "emotional dysfunction" or "emotional illness" or "affective neurosis" or melancholia or melancholias or "mood disorder*" or "mood disturbance*" or paraphrenia or paraphrenias or psychoses or psychosis or dysthymia or "dysthymic disoder*" or "mental fatigue" or "cyclothymic disorder*" or "cyclothymic personalit*" or cyclophrenia or cyclothymia or worry or apprehension or angst or anxiousness or anxiety or anxieties or fear or fears or hypervigilance or nervousness)
3. TS=(infan* or newborn* or "new born*" or perinat* or neonat* or baby or baby* or babies or toddler* or minors* or boy or boys or boyfriend or boyhood or girl* or kid or kids or child* or schoolchild* or adolescen* or juvenil* or youth* or teen* or pre-pubesc* or prepubesc* or "under* age*" or pubescen* or pediatric* or paediatric* or peadiatric* or prematur* or pre-term or preterm* or pediat* or paediat*)
4. #3 AND #2 AND #1

**Cochrane Library Search String**

diet* or "energy intake*" or "caloric intake*" or "portion size*" or "serving size*" or calorie* or cal or kcal or "caloric restriction" or "calorie restriction" or "food restriction" or "caloric intake restriction" or fasting or "calorie intake*" or "food portion*" or nutrition or nutritional or "daily recommended allowance*" or "daily recommended intake*" or "reference daily intake*" or "food guide pyramid" in Title Abstract Keyword AND depression or depressions or depressive or "affective symptom*" or "affective disorder*" or alexithymia or alexithymias or "emotional disturbance*" or "emotional disorder*" or "emotional dysfunction" or "emotional illness" or "affective neurosis" or melancholia or melancholias or "mood disorder*" or "mood disturbance*" or paraphrenia or paraphrenias or psychoses or psychosis or dysthymia or "dysthymic disoder*" or "mental fatigue" or "cyclothymic disorder*" or "cyclothymic personalit*" or cyclophrenia or cyclothymia or worry or apprehension or angst or anxiousness or anxiety or anxieties or fear or fears or hypervigilance or nervousness in Title Abstract Keyword AND infan* or newborn* or "new born*" or perinat* or neonat* or baby or baby* or babies or toddler* or minors* or boy or boys or boyfriend or boyhood or girl* or kid or kids or child* or schoolchild* or adolescen* or juvenil* or youth* or teen* or pre-pubesc* or prepubesc* or "under* age*" or pubescen* or pediatric* or paediatric* or peadiatric* or prematur* or pre-term or preterm* or pediat* or paediat* in Title Abstract Keyword

**Quality Assessment**

| **Table S1.** Quality assessment for cross-sectional studies. | | | | | | | | |
| --- | --- | --- | --- | --- | --- | --- | --- | --- |
| Study | *1. Were the criteria for inclusion in the sample clearly defined?* | *2. Were the study subjects and the setting described in detail?* | *3. Dietary intake assessed for validity?* | *4. Were confounding factors identified?* | *5. Adjustment for confounders?* | *6. Valid mental health measure?* | *7. Was appropriate statistical analysis used, and relevant data reported?* | *Score* |
| Castillo et al. (2014) | 1 | 1 | 1 | 1 | 1 | 1 | 1 | 7 |
| Dennison- Faris et al. (2017) | 0 | 1 | 0 | 1 | 1 | 1 | 1 | 5 |
| Farhangi et al. (2018) | 0 | 1 | 1 | 1 | 1 | 1 | 1 | 6 |
| Ferrer-Cascales et al. (2019) | 1 | 0 | 1 | 1 | 1 | 1 | 1 | 6 |
| Fulkerson et al. (2004) | 0 | 1 | 1 | 1 | 1 | 1 | 1 | 6 |
| Hayward et al. (2016) | 0 | 1 | 1 | 1 | 1 | 1 | 1 | 6 |
| Hoare et al. (2018) | 0 | 1 | 0 | 1 | 1 | 1 | 1 | 5 |
| Huang et al. (2018) | 1 | 1 | 0 | 1 | 1 | 1 | 1 | 6 |
| Jacka et al. (2010) | 1 | 1 | 0 | 1 | 1 | 1 | 1 | 6 |
| Khayyatzadeh et al. (2019) | 1 | 1 | 1 | 1 | 1 | 1 | 1 | 7 |
| Kohlboeck et al. (2012) | 0 | 1 | 1 | 1 | 1 | 1 | 1 | 6 |
| Kulkarni et al. (2015) | 0 | 1 | 0 | 1 | 1 | 1 | 0 | 4 |
| Oellingrath et al. (2013) | 0 | 1 | 1 | 1 | 1 | 1 | 1 | 6 |
| Puloka et al. (2017) | 0 | 1 | 0 | 1 | 1 | 1 | 1 | 5 |
| Robinson et al. (2011) | 0 | 1 | 1 | 1 | 1 | 1 | 1 | 6 |
| Sakai et al. (2017) | 1 | 1 | 1 | 1 | 1 | 1 | 1 | 7 |
| Shivappa et al. (2018) | 1 | 1 | 1 | 1 | 1 | 1 | 1 | 7 |
| Sinclair et al. (2016) | 0 | 1 | 1 | 1 | 1 | 1 | 1 | 6 |
| Tehrani et al. (2018) | 1 | 0 | 1 | 1 | 1 | 1 | 1 | 6 |
| Tanaka and Hashimoto (2019) | 0 | 1 | 0 | 1 | 1 | 1 | 1 | 5 |
| Weng et al. (2011) | 1 | 1 | 0 | 1 | 1 | 1 | 1 | 6 |
| Xu et al. (2019) | 1 | 1 | 0 | 1 | 1 | 1 | 1 | 6 |

| **Table S2.** Quality assessment for case-control studies. | | | | | | | | |
| --- | --- | --- | --- | --- | --- | --- | --- | --- |
|  | *1. Appropriate matching of cases and controls?* | *2. Were the criteria for inclusion in the sample clearly defined?* | *3. Valid dietary intake, used for both cases and controls?* | *5. Adjustment for confounders?* | *6. Valid mental health measure, for both cases and controls?* | *7. Acceptable attrition rate?* | *8. Was appropriate statistical analysis used, and relevant data reported?* | *Score* |
| Kim et al. (2015) | 1 | 1 | 1 | 1 | 1 | 1 | 1 | 8 |

| **Table S3.** Quality assessment for prospective studies. | | | | | | | | | |
| --- | --- | --- | --- | --- | --- | --- | --- | --- | --- |
|  | *1. Were the criteria for inclusion in the sample clearly defined?* | *2. Were the study subjects and the setting described in detail?* | *3. Dietary intake assessed for validity?* | *4. Were confounding factors identified?* | *5. Adjustment for confounders?* | *6. Valid mental health measure?* | *7. Acceptable attrition rate?* | *8. Was appropriate statistical analysis used, and relevant data reported?* | *Score* |
| Andersen et al. (2013) | 0 | 1 | 0 | 1 | 1 | 1 | 0 | 1 | 5 |
| Aparicio et al. (2017) | 0 | 1 | 1 | 1 | 1 | 1 | 0 | 0 | 5 |
| Jacka et al. (2013) | 1 | 1 | 0 | 1 | 1 | 1 | 0 | 1 | 6 |
| Jacka et al. (2011) | 0 | 1 | 0 | 1 | 1 | 1 | 0 | 1 | 5 |
| Michels et al. (2016) | 0 | 1 | 1 | 1 | 1 | 1 | 1 | 1 | 7 |
| Wiles et al. (2009) | 1 | 0 | 0 | 1 | 1 | 1 | 0 | 1 | 5 |
| Winpenny et al. (2018) | 1 | 1 | 1 | 1 | 1 | 1 | 0 | 1 | 7 |
| Wu et al. (2018) | 1 | 1 | 1 | 1 | 1 | 1 | 1 | 1 | 8 |
